# Unilateral Breathing in Elite Swimmers: Association With Glenohumeral Microinstability and Periscapular Fatigue

**DOI:** 10.1177/19417381261467221

**Published:** 2026-08-12

**Authors:** Elisa De Santis, Nezih Ziroglu, Gian Mario Micheloni, Andrea Giorgini, Giuseppe Porcellini

**Affiliations:** † Poliambulatorio Shoulder Team, Forlì FC, Italy; ‡ Acibadem University Atakent Hospital, Department of Orthopedics and Traumatology, Istanbul, Turkey; ^ Acibadem Mehmet Ali Aydinlar University, Vocational School of Health Services, Department of Orthopedic Prosthetics and Orthotics, Istanbul, Turkey; § University of Modena and Reggio Emilia, Department of Orthopedics and Traumatology, Sassuolo Hospital, Sassuolo, Italy

**Keywords:** periscapular muscles, rotator cuff, scapular dyskinesis, swimmer’s shoulder, trapezius

## Abstract

**Background:**

Swimmer’s shoulder is a highly prevalent, multifactorial overuse condition in competitive athletes, often involving glenohumeral microinstability, scapular dyskinesis, and muscular imbalance. Repetitive unilateral breathing patterns may create a substantial asymmetrical load, exacerbating early periscapular fatigue and contributing to symptomatic instability on the breathing side.

**Hypothesis:**

A targeted 8-week rehabilitation protocol focusing on periscapular control and rotator cuff rebalancing will result in a significant and sustained reduction in pain and improved strength symmetry in hyperlax elite swimmers presenting with shoulder pain on the breathing side.

**Study Design:**

Retrospective monocentric cohort study.

**Level of Evidence:**

Level 3.

**Methods:**

A total of 14 elite swimmers (Beighton ≥5 out of 9) with shoulder pain on the breathing side and negative magnetic resonance imaging scan for acute lesions were enrolled. Maximal voluntary isometric contraction (MVIC) strength of the upper trapezius (UT), middle trapezius (MT), and lower trapezius (LT), internal rotators (IRs), and external rotators (ERs) was measured using a handheld dynamometer at 1- and 2-year follow-up (1FU and 2FU) after an 8-week rehabilitation program. Pre- to post-treatment changes were assessed using paired Student’s *t* tests.

**Results:**

At 2FU, statistically significant and clinically relevant strength gains were observed in the MT (+24.9%; *P* = 0.002), LT (+53.6%; *P* = 0.001), IRs (+66.7%; *P* < 0.001), and ERs (+100.0%; *P* < 0.001). Mean visual analog scale pain scores decreased from 3.7 ± 1.0 at baseline to 0.85 ± 0.5 at final follow-up, corresponding to an 87.8% reduction (*P* < 0.001), indicating a durable restoration of functional shoulder balance.

**Conclusion:**

An individualized, asymmetry-aware rehabilitation program focusing on MT/LT reactivation and balanced IR/ER strengthening yields highly significant and durable functional gains and pain relief in hyperlax elite swimmers. The strength profile suggests a favorable shift away from compensatory UT activation toward optimal scapular stabilization.

**Clinical Relevance:**

Targeted scapular (MT/LT) reactivation and balanced rotator cuff rebalancing effectively reduce pain and restore functional symmetry in hyperlax elite swimmers presenting with unilateral breathing-induced microinstability.

The prevalence of shoulder pain, commonly referred to as “swimmer's shoulder,” in competitive elite athletes is strikingly high, ranging between 40% and 91% across different competitive levels.^23,30^ This spectrum of pathologies encompasses subacromial impingement, rotator cuff tendinopathy, and, particularly in the hyperlax population, glenohumeral microinstability.^1,23^ The cumulative load on the shoulder complex is immense, often involving over 30,000 cycles of repetitive overhead motion per week.^34^ This chronic microtrauma leads to characteristic adaptive changes, including increased glenohumeral laxity, altered scapular kinematics, and muscular imbalances between the rotator cuff and scapular stabilizers.^21,28^

In athletes with constitutional hyperlaxity, defined by a Beighton score of ≥5 out of 9,^1^ the intrinsic stability of the capsuloligamentous structures is reduced. This necessitates a greater reliance on the dynamic stabilization provided by the rotator cuff and periscapular musculature to maintain the humeral head centered in the glenoid fossa during the demanding overhead stroke cycle.^2,16^ Failure of this dynamic mechanism results in compensatory muscle activation patterns, early fatigue, and subsequent scapular dyskinesis.^2,28^

The act of unilateral breathing in freestyle swimming is posited as a critical, yet modifiable, risk factor that amplifies these underlying asymmetries. Biomechanical analyses demonstrate that breathing unilaterally requires greater trunk rotation and an increased scapular elevation on the breathing side.^31,32^ This asymmetrical loading leads to differential activation and premature fatigue of the scapulothoracic muscles, particularly altering the force couple between the upper trapezius (UT) and the lower trapezius (LT).^28^ This imbalance ultimately compromises scapulothoracic rhythm, a prerequisite for stable glenohumeral function.^21,27^ The cumulative effect is an increased predisposition to microinstability and pain, predominantly localized to the breathing-side shoulder.

While the efficacy of rehabilitation focusing on rotator cuff and scapular muscle rebalancing is established, few studies have assessed the durable outcomes of these protocols longitudinally, particularly in the highly specific population of hyperlax elite swimmers with confirmed unilateral breathing-side pain. This study aimed to evaluate the 2-year postintervention changes in pain and strength symmetry after an individualized, asymmetry-aware rehabilitation program. We hypothesized that a focused protocol would successfully reduce pain and restore balanced strength profiles, as evidenced by significant gains in middle trapezius (MT), LT, internal rotators (IRs), and external rotators (ER) strength and a corresponding normalization of the IR/ER strength ratio.

## Methods

### Study Design and Population

This is a retrospective monocentric cohort study that included elite swimmers treated at our institution between 2023 and 2025. All participants provided informed written consent.

#### Inclusion Criteria

- Constitutional hyperlaxity: confirmed by a Beighton 9-point score of ≥5 out of 9.^1^
- Unilateral breathing-side shoulder pain: visual analog scale (VAS) score ≥3 at baseline.
- Absence of definitive lesions on magnetic resonance imaging (MRI) scan: MRI-negative for definitive labral, full-thickness cuff tears, or significant articular cartilage pathology.
- Completion of protocol: treated with the same specific 8-week rehabilitation protocol and completed both 1-year follow-up (1FU) and 2-year follow-up (2FU) assessments.

#### Exclusion Criteria

- Patients involved in traumatic shoulder injury (e.g., dislocation, fracture).
- Signs on MRI scan of articular or full-thickness cuff lesions.
- Previous surgery or injection therapy for shoulder pathology.

A total of 14 elite competitive swimmers (8 women, 6 men; mean age 23 ± 2.5 years) were enrolled in the study.

All participants had a mean competitive swimming experience of 16.9 ± 3.1 years and were actively training at the elite level at the time of enrollment. The mean weekly training volume was 67.1 ± 14.9 km, consisting of both in-water swimming sessions and structured dryland strength training. Athletes averaged 8 swimming sessions per week and 3 dryland strength and injury-prevention sessions per week. All participants engaged regularly in conditioning and injury-prevention programs supervised by professional coaches and physiotherapists. The competitive-level distribution comprised 50% national-level and 50% international-level swimmers, with specialization in sprint, middle-distance, and long-distance freestyle events. None of the participants reported a history of shoulder surgery or acute shoulder injury within the preceding 6 months. Protocol adherence to the rehabilitation program was 100%, and the rehabilitation protocol was initiated on average approximately 1 year after symptom onset, with a range between 6 months and 1 year.

#### Demographic and Clinical Data

The dominant arm was right in 11 swimmers and left in 3. Mean competitive experience was 18 ± 2.5 years. Before the conservative treatment, all athletes completed a detailed questionnaire to record their training intensity, including hours per week spent swimming, their competition stroke, and the onset of symptoms (in years). The mean Beighton score was 6.85 ± 1.5, showing generalized hyperlaxity, with a trend toward higher scores in female swimmers. The baseline VAS pain score before the rehabilitation program was 3.7 ± 1.0. Before enrollment, 4 out of 14 swimmers (28.6%) reported being unable to finish their training sessions due to pain, and all participants claimed to have a decrease in performance during competitions.

## Testing Technique

Scapular dyskinesis and muscular strength measurements were obtained before the rehabilitation program and after completion of the 8-week protocol, with long-term clinical follow-up assessments performed at 1 and 2 years. Rotator cuff and periscapular muscle fatigue parameters were derived from repeated maximal isometric contractions recorded by the testing system. All strength assessments were performed during the in-season competitive period in a standardized clinical environment by the same experienced sports physiotherapist to minimize inter-rater variability.

### Scapular Dyskinesis Assessment

**Figure 1.**
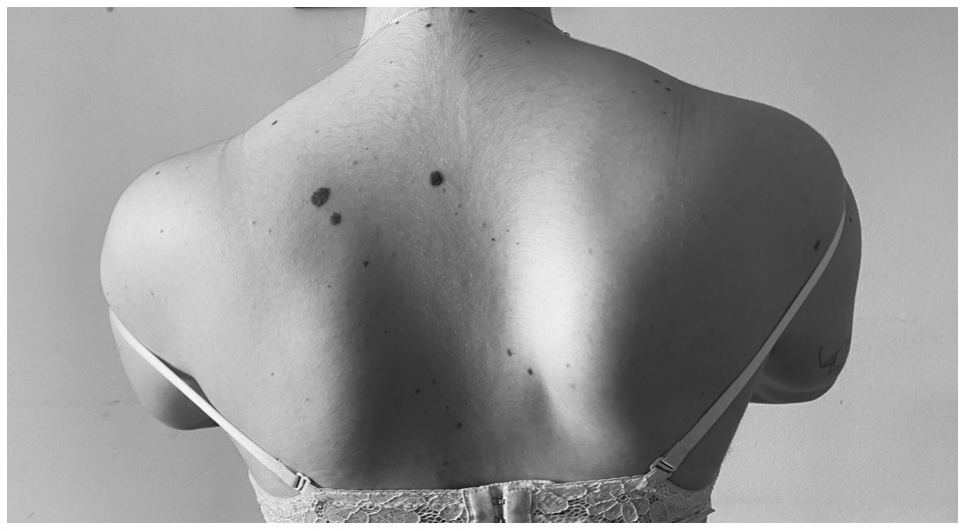
Kibler type 2 scapular dyskinesis.

Before strength testing, scapular dyskinesis patterns were evaluated and classified visually according to the scale reported by Kibler et al.^15-17^ Testing was performed by dynamic observation of the scapular motion upon arm motion, specifically by having the patients raise the arms in forward flexion to maximal elevation, and then lower them 3 to 5 times. Prominence of any aspect of the medial scapular border on the symptomatic side was recorded. Dyskinesis was classified as Type II in 11 swimmers (78.57%) and Type I in 3 swimmers (21.43%) (Figure 1).^1,15^

### Muscular Strength Assessment

Maximum maximal voluntary isometric contraction (MVIC) of the targeted muscles was performed using a Handheld Dynamometer (HHD) (Lafayette Instrument). The MVIC was recorded in kilograms and subsequently converted to Newtons for further analysis. All baseline assessments were performed during the in-season competitive period to reflect sport-specific loading conditions.

1. IR and ER strength: athletes were lying in the supine position with the arm abducted at 90 degrees in neutral rotation. The dynamometer was placed on the distal forearm.
2. Trapezius muscle strength: athletes were lying in the prone position. Three evaluations were performed with the arm at 45 degrees (UT), 90 degrees (MT), and 160 degrees (LT) of abduction, with the elbow extended and the palm facing down.

**Figure 2.**
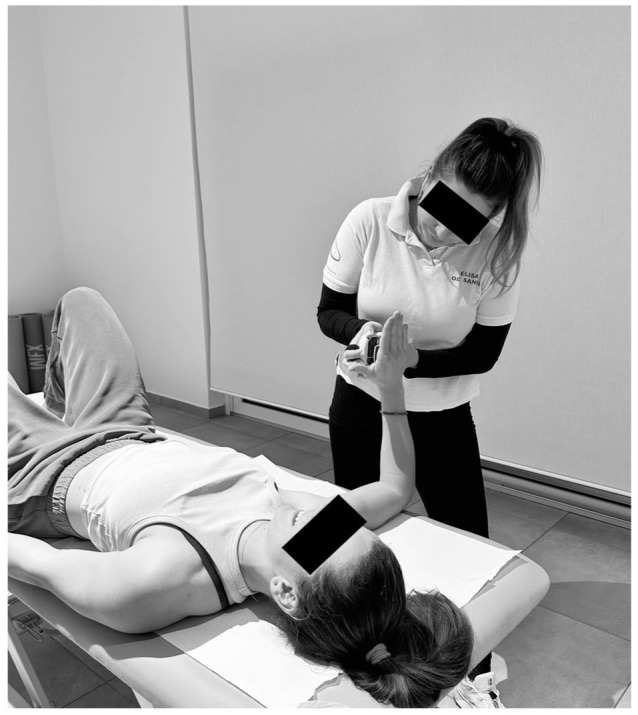
Trapezius muscle strength examination.

During the assessment, the examiner provided manual resistance by holding the HHD between their trunk and the palmar or dorsal face of the participant’s hand. For each test position, subjects performed 3 maximal isometric contractions of 5 seconds each, with a 30-second interval between them. The highest MVIC of the 3 trials was recorded (Figure 2).

### Rehabilitation Protocol

The participants were tested before and after an 8-week home-based exercise program detailed in Table 1. The 8-week duration was chosen to align with current recommendations for exercise training studies while allowing sufficient time for biological adaptation and clinical re-evaluation.^7,13,19^

**Table 1.** Exercises included in the 8-week rehabilitation program

Exercise	Sets	Repetitions/duration
Push-ups plus	3	10
Prone extension	3	10
Forward flexion in side-lying	3	10
External rotation in side-lying	3	10
Prone horizontal abduction with external rotation	3	10
I-T-Y-W exercises in the prone position	10	5-second hold
Lift-off	10	5-second hold
Internal rotation at 90° abduction	3	10
External rotation at 90° abduction	3	10

Values indicate the number of repetitions or hold duration (seconds). I-T-Y-W exercises refer to prone arm elevation movements forming the shapes of the letters I, T, Y and W to target periscapular stabilizers.

Before starting the program, the athletes were instructed in the 11 exercises by a physical therapist, and each participant was provided with a video demonstrating the correct execution. Compliance and correct form were ensured by a 3-week recheck session. Athletes performed the exercise program 4 times per week.

#### Dosing and Progression

Three sets of 10 repetitions for each exercise were prescribed, with a 45-second to 1-minute rest between sets. An additional set was performed for the pathological limb from the fourth week of training to specifically address the asymmetrical deficit. The workload was determined individually by a 10-repetition maximum (10-RM) test and progressively increased over the 2 months.

### Scapular Muscles

Appropriate scapular muscle strength and balance are critical because the scapula and humerus move together as a unit during humeral elevation.^24^

- Serratus anterior (SA): the SA works with the pectoralis minor for protraction and with the UT and LT for upward rotation. It is crucial for stabilizing the medial border and maintaining the external rotation of the inferior angle of the scapula - a key role during the recovery phase of the freestyle stroke.^5,15^ The Push-ups Plus exercise was included to selectively activate the SA.
- Trapezius (UT, MT, LT): De Mey et al^7^ revealed early MT and LT muscle activation compared with the UT during side lying external rotation, prone horizontal abduction with external rotation, and prone extension). These exercises may help restore the optimal timing of scapular muscles during dynamic activities. I-T, Y, and W exercises in the prone position were also included to improve scapular retraction.^8,25^ Asynchronous firing of the trapezius can cause superior humeral head migration, restricting the subacromial space.^23^

### Rotator Cuff Muscles

- Subscapularis: provides glenohumeral compression, stability, internal rotation, and abduction. This muscle, along with the pectoralis major and latissimus dorsi, provides major propulsion during the pull-through phase.^9^ Subscapularis fatigue may lead to unbalanced glenohumeral stabilization. The Gerber lift-off against resistance is considered an effective isolation movement.^11,29^ In addition, based on Kronberg et al,^18^ shoulder internal rotation at 90 degrees of abduction was prescribed to emphasize subscapularis activation and minimize deltoid recruitment.
- Teres minor and infraspinatus (posterior cuff): these muscles provide glenohumeral compression, external rotation, and abduction. External rotation helps clear the greater tuberosity from under the coracoacromial arch during overhead movements, reducing subacromial impingement.^9^ During the hand exit phase, ERs help the shoulder abduct and externally rotate for the next stroke. Weakness in these muscles is associated strongly with impingement syndrome. The External Rotation 90 degrees exercise was a key component of the protocol.

### Statistical Analysis

Statistical analysis was performed using R software (Version 4.2.3). Continuous variables are presented as means and standard deviations. Normality of continuous variables and paired differences was assessed using the Shapiro-Wilk test. Pre- to post-treatment changes were analyzed using paired Student’s *t* tests when normality assumptions were met.

To evaluate the localized treatment effect of the 8-week rehabilitation protocol, the change in maximal voluntary isometric contraction (delta = post – pre) in the pathological arm was compared with the change in the contralateral asymptomatic arm using an independent samples *t* test. This delta-delta comparison supported the conclusion that the observed improvements were attributable primarily to the targeted intervention rather than to generalized training adaptations.

Two-tailed *P* values < 0.05 were considered statistically significant. For each comparison, mean differences with 95% CI and standardized effect sizes (Cohen’s *d*) were reported. Effect sizes were interpreted as small (0.2), moderate (0.5), and large (≥0.8).^3,20^

A post hoc power analysis was performed for the primary outcomes using G*Power software (Heinrich-Heine University Düsseldorf).^10^ Considering the observed effect size for pain reduction (VAS), a sample size of 14 patients, and an alpha level of 0.05, the study achieved a statistical power >0.99, indicating adequate statistical power to detect the observed differences.

## Results

Pain scores decreased significantly from a baseline mean VAS of 3.7 ± 1.0 to 0.85 ± 0.5 at 2FU (*P* < 0.001), corresponding to an 87.8% sustained reduction.

**Figure 3.**
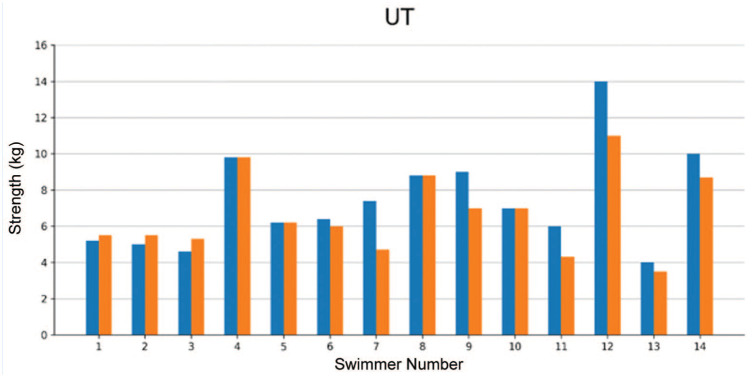
Pathological arm strength: UT. Blue, baseline; orange, postintervention. UT, upper trapezius.

**Figure 4.**
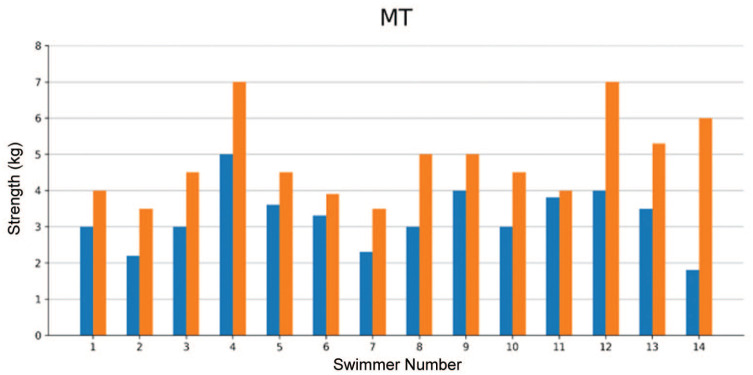
Pathological arm strength: MT. Blue, baseline; orange, postintervention. MT, middle trapezius.

**Figure 5.**
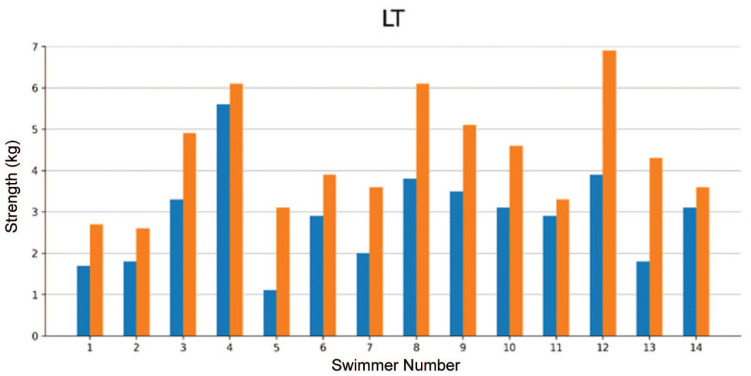
Pathological arm strength: LT. Blue, baseline; orange, postintervention. LT, lower trapezius.

**Figure 6.**
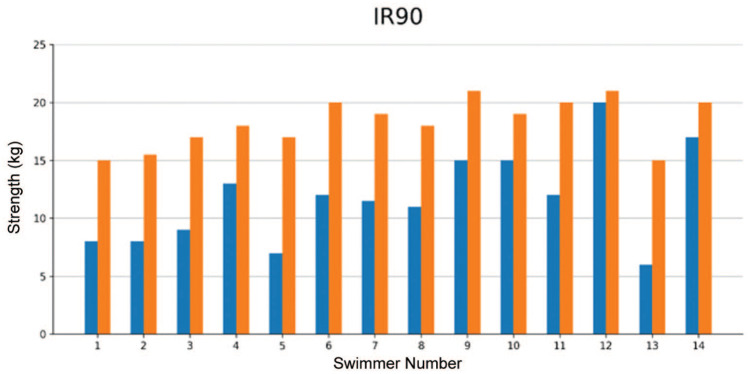
Pathological arm strength: internal rotation. Blue, baseline; orange, postintervention. IR90, internal rotation at 90 degrees.

**Figure 7.**
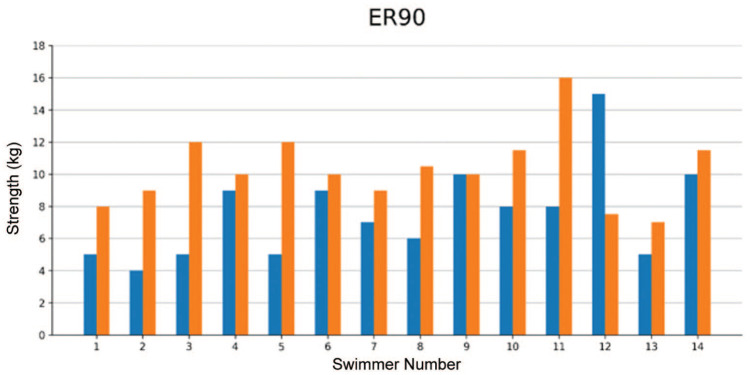
Pathological arm strength: external rotation. Blue, baseline; orange, postintervention. ER90, external rotation at 90 degrees.

Strength analysis demonstrated selective and clinically relevant improvements in the pathological arm. External rotation strength increased by 100% (8.00 ± 2.51 kg to 16.00 ± 2.78 kg; *P* < 0.001), while internal rotation increased by 66.7% (12.00 ± 2.80 kg to 20.00 ± 2.75 kg; *P* < 0.001), normalizing the internal rotation/external rotation ratio from 1.50 to 1.25. MT strength increased by 24.9% (*P* = 0.002), and LT strength increased by 53.6% (*P* = 0.001). UT strength showed a nonsignificant decrease (−5.9%; *P* = 0.334). Detailed values are presented in Figures 3 to 7.

1. Rotator cuff (glenohumeral): external rotation strength demonstrated a dramatic 100% increase (from 8.00 ± 2.51 kg to 16.00 ± 2.78 kg; *P* < 0.001), while internal rotation strength increased by 66.7% (from 12.00 ± 2.80 kg to 20.00 ± 2.75 kg; *P* < 0.001). This rebalancing corrected the asymmetrical strength ratio successfully, normalizing the internal rotation/external rotation ratio from 1.50 to 1.25.
2. Scapular stabilizers: significant, large-magnitude gains were recorded in the scapular retractors and depressors critical for stabilization: MT strength increased by 24.9% (*P* = 0.002), and LT strength increased by 53.6% (*P* = 0.001). Conversely, UT strength showed a nonsignificant mean decrease of –5.9% (*P* = 0.334), suggesting a favorable shift away from compensatory activation.

### Internal Control Analysis (Comparative Arm Data)

To establish that these improvements were a direct, specific effect of the rehabilitation and not secondary to general athletic training adaptations, the change in strength (delta: post 8-weeks – pre-rehab) of the pathological arm was compared with the change in the contralateral (healthy) arm. This analysis (summarized in Table 2) confirmed a highly significant, differential effect of the intervention.

- The strength gain in the LT of the pathological arm (delta, +1.52 kg) was statistically superior to the change observed in the contralateral arm (delta, +0.26 kg; *P* = 0.003).
- Similarly, the strength gain in the ERs of the pathological arm (delta, +8.00 kg) was statistically superior to the contralateral arm (delta, +0.43 kg; *P* < 0.001).
- Significant differential superiority for the pathological arm was also confirmed for the MT and IRs (*P* = 0.006 and *P* < 0.001, respectively).

**Table 2.** Comparative strength changes (post-pre) between pathological and contralateral arms (internal control analysis)

Muscle group	Pathological arm mean delta, kg	Contralateral arm mean delta, kg	*P* value (differential delta)
LT	+1.52	+0.26	0.003
MT	+0.95	+0.08	0.006
ERs	+8.00	+0.43	<0.001
IRs	+8.00	+0.04	<0.001
UT	-0.43	-0.32	0.79

Values represent the mean change (delta) in strength (kg) between post- and pre-intervention assessments. ER, external rotator; IR, internal rotator; LT, lower trapezius; MT, middle trapezius; UT, upper trapezius.

The absence of comparable strength changes in the asymptomatic contralateral limb provides robust internal evidence that the muscular rebalancing achieved is a direct and specific consequence of the targeted, asymmetry-aware rehabilitation protocol.

## Discussion

The findings of this 2-year follow-up study robustly support the hypothesis that a highly individualized 8-week rehabilitation protocol effectively addresses chronic, asymmetric strength deficits, and associated pain in hyperlax elite swimmers presenting with shoulder pain on the unilateral-breathing side. The most important clinical finding of this study is the demonstration of durable restoration of balanced dynamic stability, evidenced by a 100% gain in external rotation strength and a near-normalization of the internal rotation to external rotation ratio (from 1.50 to 1.25) at 2FU, which correlated directly with a sustained 87.8% reduction in mean VAS pain scores (*P* < 0.001). This provides rare longitudinal evidence of therapeutic efficacy in a unique and challenging athletic population, strengthening the case for targeted, high-resistance scapular and rotator cuff rebalancing protocols.^33^

The high prevalence of pain on the breathing-side shoulder in our cohort is a critical clinical observation that reinforces the need to view unilateral breathing as a primary, modifiable risk factor.^32^ Biomechanical and kinematic studies have indicated previously that the unilateral breathing technique induces greater trunk rotation and asymmetric scapular motion.^31,34^ Our study now provides the functional link: this asymmetric loading translates into muscular failure, as demonstrated by the strength deficits observed at baseline, which are resolved subsequently by targeted training. In the context of constitutional hyperlaxity (mean Beighton score of 6.85), the structural deficiency of the joint capsule necessitates a heightened reliance on dynamic stabilizers. When the scapulothoracic muscles are forced into an asynchronous or fatigue-prone pattern by unilateral swimming mechanics, glenohumeral microinstability is a predictable outcome.^1,5^ This outcome is consistent with the Type II scapular dyskinesis (78.57% prevalence) observed in our cohort, which is often characterized by excessive inferior-medial border prominence and altered force couples.^21^

The resolution of this dynamic instability is reflected most clearly in the differential strength changes in the periscapular stabilizers. The protocol induced significant strength gains successfully in the MT (+24.9%) and, notably, the LT (+53.6%), confirming the effectiveness of the prone position exercises (Ts, Ys, Ws) in selectively activating these crucial retractors and depressors.^7,12^ The LT is particularly essential for promoting scapular posterior tilt and upward rotation, mechanical actions that optimally clear the subacromial space during overhead motion.^21,28^ The reciprocal finding - a nonsignificant mean decrease in UT (–5.9%) strength - is highly clinically relevant. It suggests that the rehabilitation reduced successfully the compensatory over-reliance on the UT - a common pattern in scapular dyskinesis - where the UT attempts to stabilize the scapula in the absence of LT/MT efficiency.^14,22^ This shift from a compensatory UT-dominant pattern to a balanced MT/LT activation profile is arguably the most fundamental component of the observed functional recovery.^4^

Crucially, the statistical comparison with the contralateral, asymptomatic limb significantly strengthens the causal link between the intervention and the durable clinical outcomes. The internal control analysis (Table 2) demonstrated that the specific strength gains in the LT, MT, ER and IR muscles were statistically superior in the pathological arm compared with the minimal or nonexistent changes in the healthy arm. This differential improvement serves as robust evidence that the observed rebalancing is a direct and specific effect of the targeted rehabilitation protocol, mitigating the methodological limitations associated typically with uncontrolled cohort designs regarding generalized training effects or natural recovery. The localized and pronounced strength changes confirm definitively that the program corrected specifically the asymmetry profile created by unilateral swimming mechanics.

Furthermore, the strength gains in the glenohumeral rotators are key to long-term stability. The large magnitude of improvement, particularly the 100% increase in ER strength, highlights the importance of posterior cuff (infraspinatus/teres minor) rebalancing. The posterior cuff is the primary dynamic stabilizer controlling anterior humeral head translation and preventing impingement.^9^ The significant internal rotation gain (+66.7%) ensures that the subscapularis contributes adequately to joint compression during the powerful propulsive phase of the stroke.^11^ The subsequent normalization of the internal rotation to external rotation ratio from 1.50 to 1.25 addresses a widely accepted biomechanical risk factor. While swimmers are naturally internal rotation-dominant due to propulsive muscle hypertrophy, an imbalance exceeding standard ranges increases the risk of both microinstability and secondary rotator cuff pathology.^14,22,26^ Our data indicate that a 2-year sustained strength gain can correct this imbalance and mitigate symptoms effectively, supporting the necessity of ER-focused strengthening in the swimming population.

The successful implementation of an 8-week, home-based protocol 4 times per week underscores the feasibility and compliance achieved by this elite population. The protocol’s foundation, emphasizing high-resistance, low-repetition volume determined by a 10-RM test, aligns with contemporary sports medicine principles that maximal strength training is required to induce the necessary neural and morphological adaptations for long-term endurance retention and stability in deep stabilizers.^33^ The decision to re-evaluate after 2 months, rather than the standard 6 weeks, provided a more robust timeframe for adaptation in highly trained athletes.

Despite its strengths, this study has several limitations. The sample size (N = 14) is relatively small, limiting subgroup analyses and generalizability. In addition, the retrospective design lacks the methodological strength of a randomized controlled trial, although the use of an internal control partially mitigates this limitation.

The study relied on MVIC measurements, which do not capture fully the dynamic neuromuscular control required during swimming. Future studies incorporating electromyographic analysis are needed to determine whether strength gains translate into improved muscle coordination and reduced fatigue, particularly in muscles such as the serratus anterior and rhomboids.

Finally, training load variables were not recorded systematically. Weekly session frequency and the distribution of in-water versus dryland training could not be included in the analysis. Larger, prospective randomized studies are needed to better define the relationship between swimming technique, shoulder stability, and injury prevention.

## Conclusion

This 2-year follow-up study demonstrates that an individualized, 8-week rehabilitation protocol is highly effective in achieving a durable resolution of symptoms and restoration of dynamic stability in hyperlax elite swimmers presenting with shoulder pain on the unilateral breathing side.

The protocol yielded highly significant and sustained strength gains in ERs (100%), LT (53.6%), and IRs (66.7%). These gains successfully corrected the internal rotation to external rotation strength ratio from 1.50 to 1.25 and were accompanied by a substantial long-term reduction in VAS pain scores (*P* < 0.001).

These findings strongly advocate for the mandatory inclusion of targeted LT/MT activation and balanced, high-resistance rotator cuff strengthening programs to mitigate the chronic asymmetrical loading effects of unilateral breathing in competitive swimmers. Restoring this muscular balance is critical for managing glenohumeral microinstability in the hyperlax athletic shoulder.

## References

[1] Beighton P Solomon L Soskolne CL. Articular mobility in an African population. Ann Rheum Dis. 1973;32(5):413-418. doi:10.1136/ard.32.5.413 4751776 PMC1006136

[2] Borsa PA Laudner KG Sauers EL. Mobility and stability adaptations in the shoulder of the overhead athlete: a theoretical and evidence-based perspective. Sports Med. 2008;38(1):17-36. doi:10.2165/00007256-200838010-00003 18081365

[3] Cohen J. Statistical Power Analysis for the Behavioral Sciences. 2nd ed. Hillsdale, NJ: Lawrence Erlbaum Associates; 1988.

[4] Cools AM Johansson FR Borms D Maenhout A. Prevention of shoulder injuries in overhead athletes: a science-based approach. Braz J Phys Ther. 2015;19(5):331-339. doi:10.1590/bjpt-rbf.2014.0109 26537804 PMC4647145

[5] Decker MJ Hintermeister RA Faber KJ Hawkins RJ. Serratus anterior muscle activity during selected rehabilitation exercises. Am J Sports Med. 1999;27(6):784-791. doi:10.1177/03635465990270061601 10569366

[6] De Martino I Rodeo SA . The swimmer’s shoulder: multidirectional instability. Curr Rev Musculoskelet Med. 2018;11(2):167-171. doi:10.1007/s12178-018-9477-3 29679207 PMC5970120

[7] De Mey K Cagnie B Danneels LA Cools AM Van de Velde A. Trapezius muscle timing during selected shoulder rehabilitation exercises. J Orthop Sports Phys Ther. 2009;39(10):743-752. doi:10.2519/jospt.2009.3057 19801813

[8] Ekstrom RA Donatelli RA Soderberg GL. Surface electromyographic analysis of exercises for the trapezius and serratus anterior muscles. J Orthop Sports Phys Ther. 2003;33(5):247-258. doi:10.2519/jospt.2003.33.5.247 12774999

[9] Escamilla RF Yamashiro K Paulos L Andrews JR. Shoulder muscle activity and function in common shoulder rehabilitation exercises. Sports Med. 2009;39(8):663-685. doi:10.2165/00007256-200939080-00004 19769415

[10] Faul F Erdfelder E Lang AG Buchner A. G*Power 3: a flexible statistical power analysis program for the social, behavioral, and biomedical sciences. Behav Res Methods. 2007;39(2):175-191. doi:10.3758/BF03193146 17695343

[11] Greis PE Kuhn JE Schultheis J Hintermeister R Hawkins R. Validation of the lift-off test and analysis of subscapularis activity during maximal internal rotation. Am J Sports Med. 1996;24(5):589-593. doi:10.1177/036354659602400506 8883677

[12] Henning L Plummer H Oliver GD. Comparison of scapular muscle activations during three overhead throwing exercises. Int J Sports Phys Ther. 2016;11(1):108-114. 26900505 PMC4739039

[13] Hibberd EE Oyama S Spang JT Prentice W Myers JB. Effect of a 6-week strengthening program on shoulder and scapular-stabilizer strength and scapular kinematics in Division I collegiate swimmers. J Sport Rehabil. 2012;21(3):253-265. doi:10.1123/jsr.21.3.253 22387875

[14] Joo SY Kim YK. Scapular dyskinesis and associated factors in adult elite swimmers. Medicina (Kaunas). 2025;61(10):1885. doi:10.3390/medicina61101885 41155871 PMC12565977

[15] Kibler WB Sciascia A. Evaluation and management of scapular dyskinesis in overhead athletes. Curr Rev Musculoskelet Med. 2019;12(4):515-526. doi:10.1007/s12178-019-09578-5 31760624 PMC6942103

[16] Kibler WB Sciascia A Wilkes T. Scapular dyskinesis and its relation to shoulder injury. J Am Acad Orthop Surg. 2012;20(6):364-372. doi:10.5435/JAAOS-20-06-364 22661566

[17] Kibler WB Uhl TL Maddux JW Brooks PV Zeller B McMullen J. Qualitative clinical evaluation of scapular dysfunction: a reliability study. J Shoulder Elbow Surg. 2002;11(6):550-556. doi:10.1067/mse.2002.126766 12469078

[18] Kronberg M Németh G Broström LA. Muscle activity and coordination in the normal shoulder: an electromyographic study. Clin Orthop Relat Res. 1990;257:76-85. 2379377

[19] Kuhn JE. Exercise in the treatment of rotator cuff impingement: a systematic review and a synthesized evidence-based rehabilitation protocol. J Shoulder Elbow Surg. 2009;18(1):138-160. doi:10.1016/j.jse.2008.06.004 18835532

[20] Lakens D. Calculating and reporting effect sizes to facilitate cumulative science: a practical primer for *t*-tests and ANOVAs. Front Psychol. 2013;4:863. doi:10.3389/fpsyg.2013.00863 24324449 PMC3840331

[21] Ludewig PM Reynolds JF. The association of scapular kinematics and glenohumeral joint pathologies. J Orthop Sports Phys Ther. 2009;39(2):90-104. doi:10.2519/jospt.2009.2808 19194022 PMC2730194

[22] Madsen PH Bak K Jensen S Welter U. Training induces scapular dyskinesis in pain-free competitive swimmers: a reliability and observational study. Clin J Sport Med. 2011;21(2):109-113. doi:10.1097/JSM.0b013e31820f8f45 21358500

[23] Matzkin E Suslavich K Wes D. Swimmer’s shoulder: painful shoulder in the competitive swimmer. J Am Acad Orthop Surg. 2016;24(8):527-536. doi:10.5435/JAAOS-D-15-00487 27355281

[24] Mise T Kurita T Kamakari S , et al. Does scapular dysfunction alter scapular muscle activity and kinematics during swim stroke motion in adolescent swimmers? Sports Biomech. 2025;24(11):3344-3357. doi:10.1080/14763141.2024.2315257 38383332

[25] Oyama S Myers JB Wassinger CA Lephart SM. Three-dimensional scapular and clavicular kinematics and scapular muscle activity during retraction exercises. J Orthop Sports Phys Ther. 2010;40(3):169-179. doi:10.2519/jospt.2010.3009 20195020

[26] Porcellini G Ziroglu N De Santis E Micheloni GM Tarallo L Giorgini A. Midterm clinical outcomes after arthroscopic rotator cuff repair in Olympic volleyball players: return to sports and return to performance. Orthop J Sports Med. 2023;11(8):23259671231186820. doi:10.1177/23259671231186820 PMC10467408 37655246

[27] Reinold MM Escamilla RF Wilk KE. Current concepts in the scientific and clinical rationale behind exercises for glenohumeral and scapulothoracic musculature. J Orthop Sports Phys Ther. 2009;39(2):105-117. doi:10.2519/jospt.2009.2835 19194023

[28] Struyf F Tate A Kuppens K Feijen S Michener LA. Musculoskeletal dysfunctions associated with swimmers’ shoulder. Br J Sports Med. 2017;51(10):775-780. doi:10.1136/bjsports-2016-096847 28189997

[29] Suenaga N Minami A Fujisawa H. Electromyographic analysis of internal rotational motion of the shoulder in various arm positions. J Shoulder Elbow Surg. 2003;12(5):501-505. doi:10.1016/S1058-2746(03)00179-6 14564277

[30] Tate A Turner GN Knab SE Jorgensen C Strittmatter A Michener LA. Risk factors associated with shoulder pain and disability across the lifespan of competitive swimmers. J Athl Train. 2012;47(2):149-158. doi:10.4085/1062-6050-47.2.149 22488280 PMC3418126

[31] Tavares N Vilas-Boas JP Castro MA. Effect of preventive exercise programs for swimmer’s shoulder injury on rotator cuff torque and balance in competitive swimmers: a randomized controlled trial. Healthcare (Basel). 2025;13(5):538. doi:10.3390/healthcare13050538 40077099 PMC11899141

[32] Tella V Toca-Herrera JL Gallach JE Benavent J González LM Arellano R. Effect of fatigue on the intra-cycle acceleration in front crawl swimming: a time-frequency analysis. J Biomech. 2008;41(1):86-92. doi:10.1016/j.jbiomech.2007.08.006 17714719

[33] Warneke K Wagner CM Keiner M , et al. Maximal strength measurement: a critical evaluation of common methods—a narrative review. Front Sports Act Living. 2023;5:1105201. doi:10.3389/fspor.2023.1105201 36873661 PMC9981657

[34] Yanai T Hay JG. Shoulder impingement in front-crawl swimming: II. analysis of stroking technique. Med Sci Sports Exerc. 2000;32(1):30-40. doi:10.1097/00005768-200001000-00006 10647526

